# Recent Progress on the Characterization of Polymer Crystallization by Atomic Force Microscopy

**DOI:** 10.3390/polym17192692

**Published:** 2025-10-05

**Authors:** Shen Chen, Min Chen, Hanying Li

**Affiliations:** MOE Key Laboratory of Macromolecular Synthesis and Functionalization, International Research Center for X Polymers, ZJU-YST Joint Research Center for Fundamental Science, Department of Polymer Science and Engineering, Zhejiang University, Hangzhou 310027, China; chenshen@zju.edu.cn

**Keywords:** atomic force microscopy, crystalline polymer structure, crystalline polymer kinetics, crystalline polymer properties

## Abstract

The crystallization behavior of polymers affects the structure of aggregated states, which influences the properties of materials. Atomic force microscopy (AFM) is a helpful characterization tool with high spatial resolution at the nanometer-to-micrometer scale and low-destruction imaging capabilities, making it an important means of studying polymer crystallography. This review is intended for scientists in polymer materials and physics, aiming to inspire how the rich applications of AFM can be harnessed to address fundamental scientific questions in polymer crystallization. This paper reviews recent advances in polymer crystallization characterization based on AFM, focusing on its applications in visualizing hierarchical polymer crystal structures (single crystals, spherulites, dendritic crystals, and shish kebab crystals), investigating crystallization kinetics (in situ monitoring of crystal growth), and analyzing structure–property relationships (structural changes under temperature and stress). Finally, we introduce the application of the latest AFM technology in addressing key issues in polymer crystallization, such as single-molecule force spectroscopy (SMFS) and atomic force microscopy–infrared spectroscopy (AFM-IR). As AFM technology advances toward higher precision, greater efficiency, and increased functionality, it is expected to deliver more exciting developments in the field of polymer crystallization.

## 1. Introduction

The crystalline state, as a fundamental component of the aggregated structure of polymers, profoundly influences the properties of polymer materials [1,2]. The unique long-chain structure of polymers causes them to exhibit crystallization behavior that differs from that of small molecules, resulting in specific physical and chemical properties [3,4,5,6]. These properties enable crystalline polymer materials to be widely used in fields such as materials [7], biology [8], industry [9], and agriculture [10]. For crystalline polymers, phase separation during processing and structure evolution during aging and under external fields significantly affect material performance. Thus, a suitable technique for revealing the structure of materials ex situ or in situ would be helpful to understand these materials and establish a fundamental understanding of the materials’ structure and performance.

In early years, researchers used methods such as X-ray diffraction (XRD) [11,12,13] and differential scanning calorimetry (DSC) [13,14,15] to characterize the microstructure of crystalline polymers. Although these characterization methods provided information on crystallinity, phase transformation, and crystallization rate at the macroscale, they had obvious shortcomings in terms of micro-area characterization. With the development of optical microscopy (OM) and electron microscopy (EM) technologies, the microscopic structure of polymer crystals has been further revealed. However, the spatial resolution of optical microscopes limits the observation of structures at the smaller micrometer-to-nanometer scale. Electron microscopes, on the other hand, are limited in their further application due to the low conductivity and weak electron beam tolerance of polymer materials.

Atomic force microscopy (AFM), as a non-destructive, highly sensitive nanoscale characterization technique, has become an important tool for studying the crystalline morphology of polymers [2,16,17,18]. Table 1 compares its advantages and limitations with other characterization methods to further highlight why AFM stands out. AFM can provide information on the surface morphology, roughness, crystal orientation, and physical properties of polymer crystals. Additionally, AFM can be combined with other equipment and methods, such as scanning electron microscopes [19], spectroscopic testing [20], and nanoindentation techniques [21], to achieve complementary characterization results.

Although several reviews on AFM in polymer science exist, most are presented from the perspective of AFM technology, and few focus specifically on polymer crystallization behavior. This review adopts a polymer crystallization behavior perspective to explore what AFM can achieve in this field and how it influences our understanding of polymer crystallization. First, we introduce the characterization of polymer hierarchical crystal structures (from molecular arrangements to crystalline textures) by AFM and highlight its advantages. Second, we discuss the application of AFM for in situ monitoring of polymer crystal growth processes and elaborate on how AFM results validate and supplement crystallization kinetics theory. Third, we present the use of AFM to measure the physical properties of polymer crystals, focusing primarily on its application in testing under thermal and tensile conditions and establishing correlations between the properties and crystal structures. In addition, we introduce two new AFM techniques, single-molecule force spectroscopy (SMFS) and AFM–infrared spectroscopy (AFM-IR), and discuss their applications as well as prospects in polymer crystallization characterization. Finally, we summarize the recent challenges and limitations of AFM technology in polymer crystallization characterization and outline future development directions. By fully tapping into the potential of AFM technology, we can gain further insights into the complexity of polymer crystallization behavior, providing new methods and ideas for the design and development of high-performance polymer materials.

## 2. Basic Principles of AFM Technology

**Figure 1:**
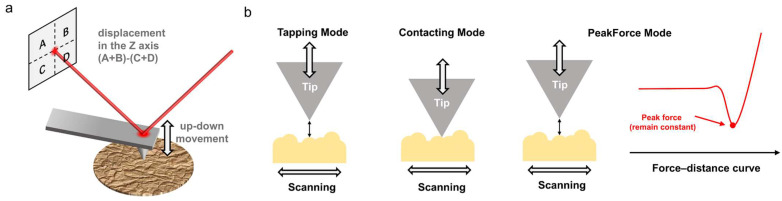
Illustration of AFM working principle. (**a**) Schematic illustration of probe disturbance determined by laser reflection. (**b**) Three commonly used modes of AFM in the field of polymers.

The operating principle of AFM is to achieve nanoscale imaging by detecting the interaction forces between the tip of the probe and the sample surface (such as van der Waals forces, electrostatic forces, magnetic forces, etc.) [22]. The probe is installed at the end of a flexible cantilever. When it approaches the sample, the interaction force causes the cantilever to shift or vibrate. The minute changes in the probe are usually detected by optical methods [17]. By shining a laser onto the cantilever of the probe and using a four-quadrant detector to detect changes in the position of the reflected light, the position of the probe can be determined (Figure 1a).

Atomic force microscopes are known for their versatility. By switching between different operating modes, researchers can obtain multidimensional information on surface morphology, mechanical properties, electrical properties, and other characteristics. In the field of polymers, three modes are commonly used: tapping mode, contact mode and PeakForce mode (Figure 1b) [2].

- Tapping mode: The probe periodically approaches the sample surface at its resonant frequency. As it nears the sample, the probe undergoes compressed oscillation due to the interaction forces, causing changes in amplitude. These changes are captured by a four-quadrant detector through the reflected laser and converted into surface topography information via post-processing. Since the probe does not contact with the sample surface, there is no risk of damage to the sample, making it suitable for studying soft or fragile samples, such as thin film surfaces and molecular-scale images. This is particularly important for in situ observation of the crystallization process in polymer films and for characterizing the structure of polymer crystals at the molecular scale. By changing the setpoint and drive amplitude of the probe, it is possible to select imaging based on intermolecular attractive forces or repulsive forces [23].
- Contact mode: As the probe glides across the surface while maintaining contact between the tip and the sample, changes in the sample’s topography cause vertical deflection of the cantilever. This results in the position of the reflected laser changing. By measuring the positional shifts in the reflected laser corresponding to each scanning point through the four-quadrant detector, information about the sample’s surface topography is obtained. This mode offers high sensitivity and resolution but can easily damage soft samples. Many AFM functionalization tests are based on contact mode, such as lateral force microscopy (LFM), piezo force microscopy (PFM), etc. [24]. These are widely used for testing the physical properties of polymer crystals, which help researchers to establish relationships between structure and properties.
- PeakForce mode: The principle behind PeakForce mode is relatively complex, so only a brief introduction is provided here. Further details can be found in Ref. [25]. The peak force between the probe and sample is maintained constant (by moving the scanning tube). When scanning along the X and Y axes, the detector’s movement in the Z direction is recorded to obtain the surface topography image of the sample and nanomechanical information. Unlike conventional tapping mode, this approach employs a cantilever oscillating at a non-resonant frequency. Under these conditions, each interaction between the tip and the sample can be measured, yielding a continuous force–distance curve. This mode is crucial for measuring the mechanical and electronical properties of polymer crystals under pressure [26].

Additionally, there exists a non-contact imaging mode utilizing van der Waals forces. In this mode, the probe remains at a greater distance from the sample, resulting in minimal damage [27], but issues such as slow scanning speed and poor imaging quality have limited its application in the field of polymer crystallization. Nevertheless, current research has demonstrated that non-contact AFM possesses unique advantages in high-resolution imaging of organic small molecules and oligomer crystals [28] and may potentially extend to polymer crystal systems in the future.

Once the position signal from the probe has been captured by the quadrant detector, it is filtered and demodulated to generate valid data [29]. These data typically provide a variety of information regarding polymer crystallography. Height, amplitude (in tapping mode), and deflection (in contact mode) images, which use changes in the Z-axis position of the probe for imaging, typically reveal information about polymer crystal morphology and roughness [30]. Phase imaging, as an extension technique in tapping mode, utilizes phase shifts induced by factors such as viscous forces and friction to generate images. These factors typically stem from differences in composition and molecular arrangement [31]. Consequently, phase differences can be employed to visualize polymer composition distribution, crystalline domain distribution, and other structural information [32]. In addition, information on the interaction between the probe and the sample (such as force, current, etc.) can also be used for localized analysis of the physical properties of polymer crystals.

However, AFM also has some limitations. First, AFM imaging speed is relatively slow. Second, AFM typically requires the sample surface to be clean and relatively flat (with irregularities preferably less than 1 μm). Third, the limited scanning size of AFM (maximum 150 × 150 μm) makes it unsuitable for imaging large-scale samples. In addition, the operating functional mode of AFM is relatively complex and requires experience. Overcoming these limitations is one of the trends in the development of AFM technology. To overcome the disadvantage of slow imaging speeds, high-speed AFM emerged. In this technique, various components, including the cantilever, electronic circuitry, sample stage scanner, and cantilever deflection detection system, are optimized to achieve high-speed performance (maximum 20 frames/s) [33]. As scanning speed increases, expanding the scanning range also becomes possible [34]. In addition, the integration of AFM with other characterization techniques, as mentioned above, also represents a development trend for AFM [35].

## 3. Characterization of Polymer Crystal Structures

Polymer crystallization is the result of ordered stacking of molecular chains. The arrangement of these ordered structures forms complex hierarchical polymer crystalline textures, such as single crystals, spherulites, dendritic crystals, and shish-kebab crystals. These crystal structures were first discovered under OM or EM and were later visualized more clearly by AFM.

**Figure 2:**
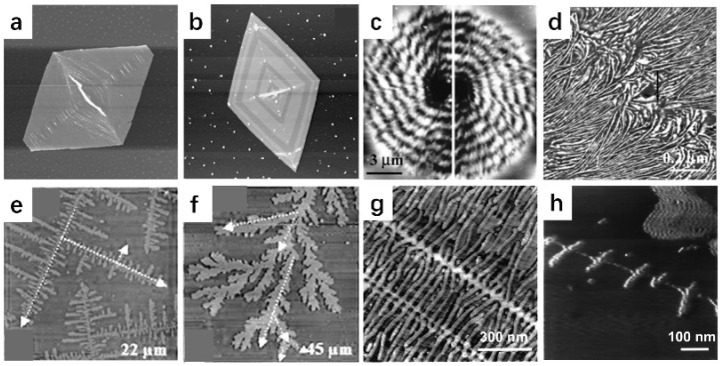
AFM imaging of different crystalline textures in polymers. (**a**) A truncated lozenge-shaped PE single crystal. Scanning size is 10 μm × 10 μm; height scale is 100 nm. (**b**) Internal distribution of different thicknesses of the PE single crystal. Scanning size is 20 μm × 20 μm; height scale is 40 nm. Reproduced with permission [36]. Copyright 2004, American Chemical Society. (**c**) AFM height image of the entire spherulite of PHB-co-HHx. (**d**) Phase image showing lamellar morphology near the bands of the spherulite. Reproduced with permission [38]. Copyright 2004, American Chemical Society. (**e**) AFM height image of dendritic PEO crystals (crystallized at 47.5 °C). The height range is 60 nm. (**f**) AFM height image of seaweed-like PEO crystals (crystallized at 25 °C). The height range is 100 nm. Reproduced with permission [39]. Copyright 2006, American Chemical Society. (**g**) AFM phase image of PE shish kebab structure taken at room temperature and (**h**) at 135 °C. Reproduced with permission [40]. Copyright 2006, American Chemical Society. In all of the above figures, the lightness and darkness of the colors represent the large and small values of height (or phase), respectively.

Figure 2a shows a height image of a PE single crystal taken by AFM [36]. The single crystal was prepared in solution by using the temperature-controlled self-seeding method and then was transferred onto the substrates. The results of the thickness were similar to those obtained by electron microscopy [37], both of which were much smaller than the full length of the polymer chain, further proving that chain folding occurred during the crystallization process of the polymer. Figure 2b shows the unique advantages of AFM in imaging polymer single crystal structures. Tian et al. changed the crystallization temperature during the crystal growth, resulting in distinct thickness steps within the crystal [36]. This minute difference in thickness (approximately 2 nm) matched the z-axis resolution of the AFM perfectly, allowing it to be clearly captured in the AFM height image.

Figure 2c shows the surface topography of a poly(R-3-hydroxybutyrate-co-R-3-hydroxyhexanoate) (PHB-co-HHx) random copolymer banded spherulite isothermally crystallized at 45 °C [38]. The average band spacing was the same as that observed by polarized optical microscopy (POM). Figure 2d shows the lamellar morphology near the concentric extinction bands. Notably, due to the relatively large surface undulations of the spherulite thin film, the phase image better reflects the microstructure inside the spherulite than the height image taken by AFM.

Zhai et al. obtained dendritic (Figure 2e) and seaweed (Figure 2f) structures in low-molecular-weight polyethylene oxide (PEO) monolayers by changing the crystallization temperature [39]. At room temperature, the 90° angle between the main and side branches indicated that the crystals grew with strong anisotropy. As the crystallization temperature increased, the angle between the main branch and the side branch tended to be 45°, indicating that the anisotropy of crystal growth weakened, forming a seaweed-like crystal morphology.

Hobbs et al. used AFM to study the crystallization of PE shish kebab crystals [40]. Figure 2g shows a phase image of a PE shish kebab structure formed by shearing a sample with a razor blade, taken at room temperature. The image reveals the presence of individual lamellae, along with three distinct rows of nuclei which extend diagonally across the field of view. Figure 2h shows the results of PE shish kebab crystal crystallization at 135 °C, where the distribution of the shish kebab structure is sparser due to the reduction in nucleation density. This morphology is same as that seen in crystallization in stirred solutions [41].

AFM also has the capability to reveal structure information at the atomic scale. Before the advent of AFM, researchers attempted to image lattice-scale structures by using scanning tunneling microscopy (STM). However, in the field of polymers, the application of STM was limited to conductive polymers [42,43,44] and molecular monolayers adsorbed on flat conductive substrates [45,46]. The appearance of AFM promoted ultra-high-resolution imaging of insulating polymers [47].

**Figure 3:**
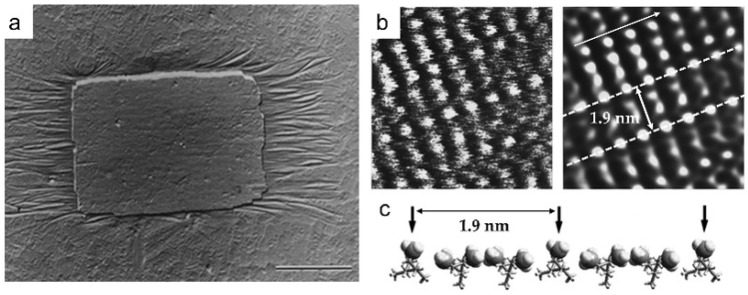
Characterization of the β phase of i-PP. (**a**) Transmission electron microscope bright-field image of the i-PP β phase of thin film epitaxially grown on a DCHT substrate. Scale bar: 5 μm. (**b**) HRAFM images of the i-PP β phase. The two images on the left and right represent the results before and after Fourier filtering. Size of imaged area:  12.5 nm × 12.5 nm. The chain direction is indicated by the arrow in the upper left corner. The 1.9 nm period corresponds to the distance between two adjacent chains. (**c**) Conformation of β-iPP molecular chains in the c-axis direction. Reproduced with permission [54]. Copyright 1998, American Chemical Society.

Isotactic polypropylene (i-PP) has α and β phases of crystal structures, where the β phase is metastable compared to the α phase and has a faster growth rate at high temperatures [48,49,50]. However, after the β phase was first reported by Keith in 1959 [51], its structure remained unknown for 35 years [52,53]. The reason was that α and β phases coexist at high temperatures, making it difficult to obtain the pure β phase. Stocker et al. successfully imaged the chain structure of the β phase by using high-resolution atomic force microscope (HRAFM) technology [54], which provided the first real-space illustration of frustration in polymer crystallography. They epitaxially grew a thin film of the i-PP β phase on the substrate of N,N′-dicyclohexylterephthalamide (DCHT) crystal (Figure 3a). Subsequently, they analyzed the TEM results to determine that the (110) plane of the β phase was the epitaxially grown face and further characterized the molecular chain arrangement on the (110) surface by HRAFM. Here, in order to reduce signal noise, they chose to perform scanning in liquid isopropanol. Figure 3b shows two HRAFM images corresponding to the possible conformations of the i-PP molecule in the β phase, indicating that it was a helical structure with a period of 1.9 nm (Figure 3c).

To obtain reliable HRAFM images, test samples must meet the following requirements. First, the surface of the sample must remain stable during scanning. Second, it must be molecularly flat within the scanning range. Third, the surface of the sample must be ordered [47]. In the case of organic small-molecule systems, the surface of single crystals can meet the above requirements [55,56]. HRAFM is often utilized to characterize crystal structures that are challenging to investigate using transmission electron microscopy (TEM), owing to their sensitivity to the electron beam. In polymer systems, oriented films [57,58], fibers [59], and extended-chain single crystals [47,60,61,62] are typically available to provide suitable areas for HRAFM testing. However, high-resolution imaging of the surface of polymer folded-chain lamellar crystals has not yet been achieved. The reason may be that molecular segments in the folded chain structure undergo movement during scanning, causing the surface to become unstable, or that the surface of the folded chain is not sufficiently ordered.

**Figure 4:**
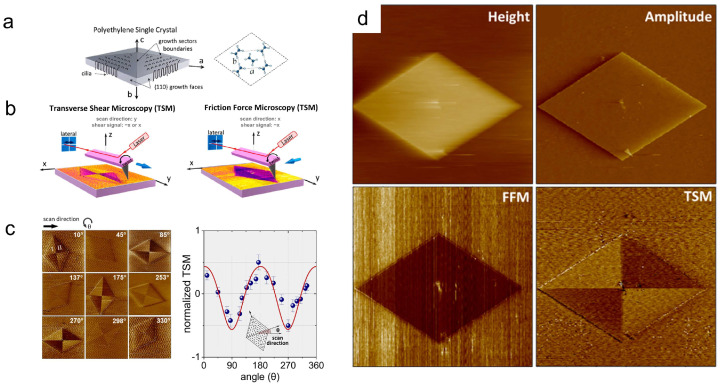
Sector visualization of polymer single crystals using AFM. (**a**) Schematic of PE single crystal sector structure. (**b**) Comparison of image results when scanning parallel and perpendicular to the axis of the cantilever. (**c**) Visualization of PE single crystal sector images obtained by counterclockwise rotation of the crystal. The right figure shows the curve of the difference between two adjacent sectors (signals in sector I minus those of sector II) as a function of rotation angle (data normalized). (**d**) Microscopic images of PE single crystals in imaging of different channels in lateral force mode. Image size, 30 × 30 μm^2^; scan rate, 2.45 Hz; and normal load, 2 nN. Reproduced with permission [67]. Copyright 2022, American Chemical Society.

While AFM struggles to directly image chain-folded surfaces at the atomic scale, it can image the orientation of chain folds. In polymer lamellar crystals, the chain folding direction is parallel to the growth front, forming different sectors (Figure 4a) [63,64,65]. Wittmann et al. modified the surface of lamellar crystal by vapor deposition of short-chain polyethylene, confirming the existence of sectors [66]. In recent years, Hamidinejad et al. directly visualized the sector structure through AFM transverse shear microscopy (TSM) [67]. When the probe passes over the folding surface, the chain folding structure provides a shear force to the probe, causing the cantilever to deflect. As chains fold in different directions, the direction and magnitude of shear forces vary, resulting in different contrasts in lateral force mapping across different sectors (Figure 4d). It is worth noting that the contrast differences between different sectors are related to the scanning direction of the probe. Higher sensitivity can be achieved when the scanning direction is parallel to the cantilever axis. (Figure 4b). Figure 4c shows that the signal contrast is also related to the angle between the scanning direction and the single crystal orientation, exhibiting a clear 180° periodic dependence.

**Figure 5:**
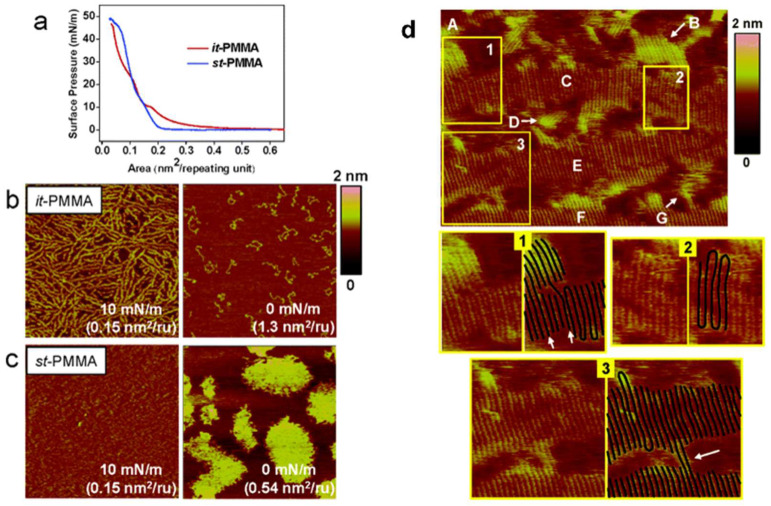
Preparation of LB crystalline films and AFM imaging. (**a**) Surface pressure–area curve of it-PMMA and st-PMMA on water. (**b**) AFM height images of the monolayers of it-PMMA and (**c**) of st-PMMA. Scale = 1 × 1 μm^2^. (**d**) High-magnification AFM height image of it-PMMA deposited on mica at 10 mN/m. Scale = 100 × 75 nm^2^. Reproduced with permission [69]. Copyright 2005, American Chemical Society.

Although the thickness and presence of sectors provide evidence of chain folding during polymer crystallization, it has been difficult for researchers to observe chain folding structure directly. AFM is considered to be the key to observing polymer chains at the molecular level and has been widely studied. However, in most systems, attempts to directly image molecular chains has failed. This is mainly because most surfaces do not have molecular-level flatness, and the disturbance of the probe during scanning causes the molecular chains to move, reducing the quality of the image. Langmuir–Blodgett (LB) monolayer films are recognized as excellent systems for studying polymer structures at the molecular level [68]. Since LB films require molecules to arrange themselves into a monolayer at the water–gas interface, the selected polymer must be amphipathic. The monolayer can be transferred onto a solid substrate by controlling the surface pressure and the substrate’s movement. Kumaki et al. prepared LB films of isotactic poly(methyl methacrylate) (it-PMMA) and syndiotactic poly(methyl methacrylate) (st-PMMA) by the vertical dipping method and determined that crystallization had occurred based on the surface pressure–area curve (Figure 5a) [69]. AFM images clearly show that as surface pressure increased, independent it-PMMA molecular chains became continuous films, ultimately forming lamellar crystals at a pressure of 10 mN/m (Figure 5b). st-PMMA, even in a dilute state, exhibited an aggregated structure, which then formed a continuous film under compression (Figure 5c). This is also why the curve for st-PMMA in Figure 5a has one less plateau compared to that of it-PMMA. Viewing the LB crystal film of it-PMMA at a higher magnification allows the chain folding structure to be clearly observed, as well as the intercalated molecules between the lamellar crystals and other defects (Figure 5d).

Here, it should be noted that although traditional thinking holds that tapping-mode resolution is inferior to contact mode, resolution slightly better than 1 nm can be achieved on LB films by tapping mode [68]. Recently, Mullin and Hobbs used torsional tapping-mode AFM (TTAFM) to further improve resolution and successfully observed molecular images of polyethylene molecules [58]. They utilized T-shaped cantilevers with laterally offset tips to enhance the resolution of AFM [70], since torsionally oscillating cantilevers exhibit higher resonant frequencies and quality factors compared with flexurally oscillating cantilevers [71,72]. Advances in AFM technology will enable us to gain a deeper and more detailed understanding of polymer crystal structures.

## 4. Characterization of Polymer Crystallization Kinetics

Another feature of AFM is that, depending on the instrument accessories, measurement can be carried out under various environments, such as air, inert gas, vacuum, liquid phases, and variable temperature conditions, providing a powerful tool for observation of polymer crystal growth. Furthermore, AFM imaging is non-destructive in most cases, making it possible to monitor crystal growth processes in real time. Therefore, researchers can verify existing crystallization kinetics theories and propose new hypotheses by observing the structural evolution of polymers during the crystallization process in situ.

**Figure 6:**
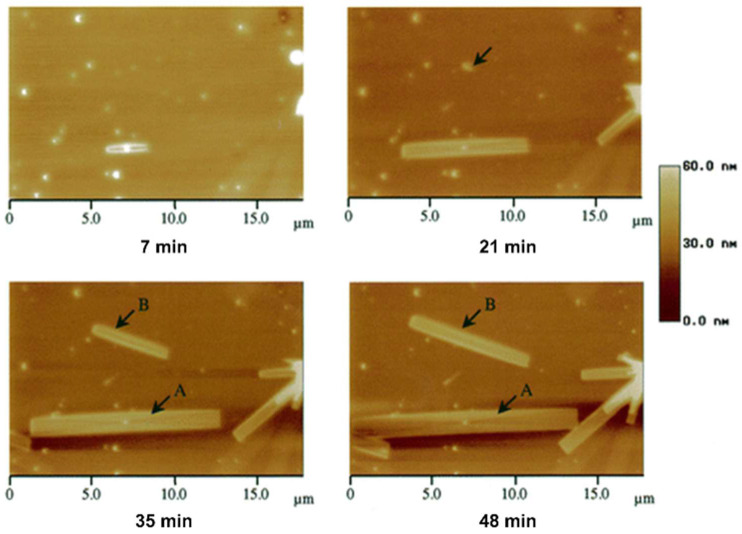
A set of AFM height images of s-PP lamellar crystals grown from melt films at 120 °C at different times. Reproduced with permission [73]. Copyright 2000, American Chemical Society.

Polymer crystallization is a typical first-order phase transition process. The formation of crystals from a disordered phase requires nucleation before growth can occur. Zhou et al. observed the growth process of lamellar crystal in syndiotactic polypropylene (s-PP) films by coupling AFM with a hot stage (Figure 6) [73]. At 7 min, a small single crystal first formed at the nucleation point in the image, and then, as the crystallization time increased, the size of the lamellar crystal became larger. During growth, the crystal maintained an aspect ratio of 10, indicating strong anisotropy in the growth rate of different crystal facets. In addition, they also found that during the early stages of growth (7~21 min), the growth rates of the crystal surface were almost constant, which has also been observed in other polymer crystallization systems [74,75].

Meanwhile, they also found that there were differences in the thickening process of different sectors during growth. As the crystallization process progressed, the thickness of the thicker (100) sector gradually stabilized, while the thinner (010) sector continued to thicken. In other words, the difference in thickness between the two sectors continued to decrease.

In addition to the lamellar crystal system, the formation process of hierarchical polymer crystalline texture is also of great interest in polymer crystallization research. Barham et al. used AFM to measure the growth rates of poly(hydroxybutyrate-co-valerate) spherulites and internal lamellar crystals, finding that the growth rate of individual lamellar crystals within spherulites differs from the overall growth rate [76]. Specifically, although the overall growth rate remains constant, the growth rate of individual lamellar crystals does not.

**Figure 7:**
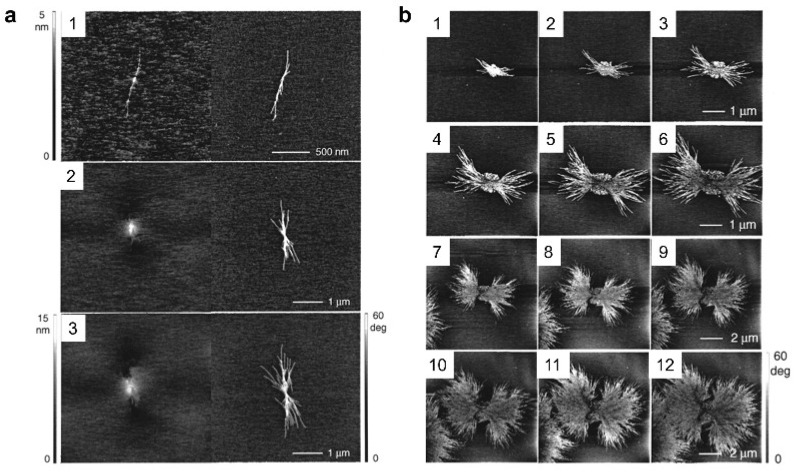
The whole process of PBA-C8 spherulite formation. (**a**) Development of a lamellar sheaf. Left: height image. Right: phase image. 1–lamellae are bred from a single nucleus; 2–the lamellae splaying apart and breeding more lamellae; 3–formation of a lamellar sheaf. (**b**) A series of AFM phase images showing the formation of a spherulite. 1–12: corresponding crystallization time from short to long. Reproduced with permission [77]. Copyright 2001, American Chemical Society.

Li et al. used in situ AFM imaging to observe the formation process of polymer spherulites [77]. In this study, they selected poly(bisphenol A octane ether) (PBA-C8) as the subject of their research because this polymer has a crystallization rate that matches the imaging rate of AFM. In the early stages of growth, individual lamellar crystals triggered the formation of additional lamellar crystals. As these primary crystals grew at both ends and diverged from each other, they produced more crystals through secondary nucleation. These crystals eventually evolved into a bundle of lamellar crystals (Figure 7a). As the lamellar crystals continued to grow, the bundle of lamellar crystals gradually developed into the skeleton of the spherulite. Additionally, it was observed that the size of the symmetrical eye at the center of the spherulites increased over time (Figure 7b). At the same time, they found that the growth front of the spherulite was not as smooth as observed in OM images, and the shape of the spherulite only became apparent at the end of crystallization.

Whereas in previous discussions we considered the influence of AFM probes on in situ crystallization observations to be negligible, in this work, they found that changing the setpoint amplitude ratio of the probe affected the orientation of lamellar crystals during growth. As the setpoint amplitude ratio decreased, the probe moved closer to the sample surface, increasing the pressure exerted on the surface. When the setpoint amplitude ratio was 0.72, the probe had little disturbance on the sample, and the lamellar crystals grew along the preferred edge-on orientation. However, when the value decreased to 0.44, the orientation of the lamellar crystals changed from edge-on to flat-on, possibly because the “a-b” face of the lamellar crystals was more stable under the pressure of the probe. This warns us that AFM may also provide some false information in some cases. From another perspective, we can also use AFM probes to regulate the polymer crystallization process. Liu et al. recently managed to control the growth kinetics of polymer crystallization by raster scanning the tip coated with poly-DL-lysine hydrobromide (PLH) across the substrate [78]. They found that the main function of the tip is to control the position of crystallization and the concentration of PLH.

**Figure 8:**
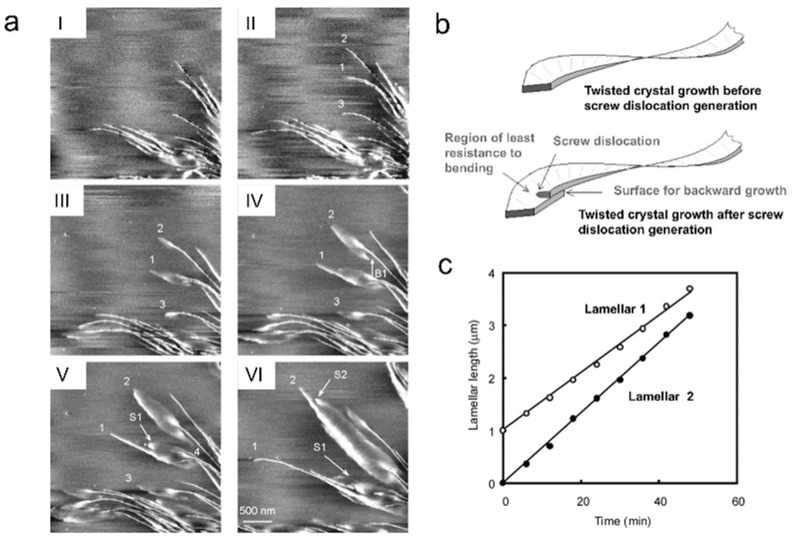
Observation of the lamella growth process in spherulites. (**a**) Real-time AFM phase images showing lamellar twisting process at 75 °C. The images in I–VI correspond to elapsed time of 6, 12, 18, 24, and 36 min, respectively. (**b**) Model of the effects of a giant screw dislocation on crystal morphology. (**c**) Length change curves of lamellae 1 and 2 over time. Reproduced with permission [38]. Copyright 2004, American Chemical Society.

Xu et al. studied phenomena such as twisting, bending, backward growth, screw dislocations, etc., in lamellar crystals during their growth process within PHB-co-HHx spherulites by real-time AFM observation [38]. Figure 8a shows how a single lamellar crystal changed its orientation during growth. When edge-on lamellae grew to approximately 2 μm, they bent counterclockwise in the tangential direction and twisted into flat-on lamellae. Flat-on lamellar crystals appeared lozenge-shaped and grew forward and backward, as shown by arrow B1 in Figure 8a IV. Concurrently, screw dislocations appeared behind the growth tip, resulting in new lamellar crystals forming on both sides of the leading edge, indicated by arrows S1 and S2. The researchers believed that screw dislocations created conditions for the backward growth of lamellar crystals and reduced the cross-section for twisting (Figure 8b). However, they further discovered that twisting could occur even in positions far away from any screw dislocations, indicating that screw dislocations were not a necessary condition for causing lamella twisting. They also recorded the growth rates of lamellae 1 and 2, finding that they remained constant (Figure 8c).

In summary, the main applications of in situ AFM for polymer crystallization research are to monitor growth rates and observe structural evolution. However, due to the relatively slow imaging rate of AFM, it is incompatible with some fast crystallization systems. Currently, AFM with faster scanning speeds has been developed [79,80] and has found some application in crystal growth [81], but the fast scanning also indirectly increases the load on the probe, which may produce some false signals. Hence, overcoming the contradiction between imaging quality and scanning speed is a challenge for AFM to achieve wider application in the in situ observation of crystallization processes.

## 5. Characterization of the Structure–Property Relationship

Corresponding to crystallization kinetics, AFM can also study the response of polymer crystal structures to external conditions (such as temperature, humidity, electric fields, etc.). In situ observation of structural changes in crystals under external field conditions is of great significance for understanding the structure–property relationship. Here, we mainly discuss the structural changes that occur in polymer crystalline structures under the influence of heat and force and attempt to establish a connection with the corresponding physical properties.

When the temperature exceeds the melting point, crystals undergo the reverse process of crystallization melting. However, most polymer crystals are metastable structures, and during heating, processes such as lamella thickening and crystal structure transformation occur, making the melting process of polymers more complex [82,83]. Also, for the same polymer, there is a significant difference in melting point between polymer crystals with different structures [84]. These different structures can often be combined in one crystalline polymer system because of the complexity of polymer crystallization. In melting point tests such as DSC, the melting point is the average information of the whole sample, whereas in situ temperature-controlled AFM allows us to observe the different melting points corresponding to different structures within the polymer system. To accurately measure the melting point, AFM is equipped with a high-precision temperature control module that simultaneously controls the temperature of the sample and the tips. The feedback from the thermocouple under the sample stage ensures that the actual temperature of the sample reaches the set value.

**Figure 9:**
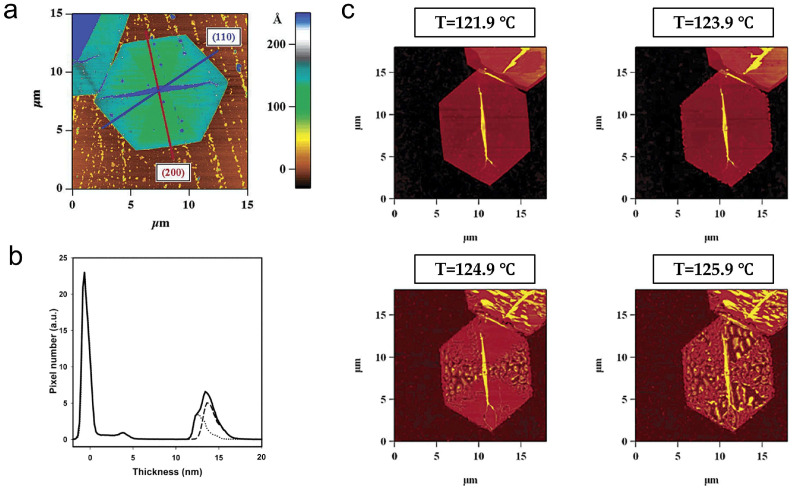
Morphology and melting of PE single crystals. (**a**) Height image of PE truncated single crystal. (**b**) AFM height histograms, including four (110) and two (200) sectors (full line); histograms of the (110) sector (dotted line) and of the (200) sector (dashed line). (**c**) AFM images of single crystals at different temperatures. Reproduced with permission [85]. Copyright 2003, American Chemical Society.

Hocquet et al. used AFM to observe the morphology of PE single crystals (Figure 9a) and their melting process at high temperatures, finding that the thinner (200) sectors had lower melting points (Figure 9b,c) [85]. This phenomenon has also been observed in the i-PP system [73]. Based on the Gibbs–Thomson relationship, they calculated the surface free energies of the (110) and (200) sectors to be 58 and 56 erg/cm^2^, respectively. Interestingly, by combining data from Raman spectra at low shifts, they revealed that different sectors had similar stem lengths, and that differences in thickness were caused by inconsistent molecular chain tilt angles. In other words, even if the crystal zone length is the same, the magnitude of the tilt angle will affect the melting point of the crystal. Simultaneously, they also discovered a process of lamella thickening during melting, with the thickest areas reaching twice the initial value.

However, Organ et al. discovered that no obvious sector-preferential melting was observed in AFM at lower heating rates [83]. Therefore, they speculated that subtle changes occurred within the crystal at temperatures significantly below the melting temperature, thereby affecting subsequent melting behavior. They observed shape distortion in the crystal before it melted, leading them to infer that this subtle change might have been caused by stress resulting from thermal expansion. Unfortunately, different researchers using AFM to observe the melting of PE single crystals have obtained different experimental results, making it difficult to discuss the melting behavior. Thermal history [86], molecular weight [87], crystal shape [88], and substrate [89] may all be the factors influencing the thermal stability of polymer crystals.

**Figure 10:**
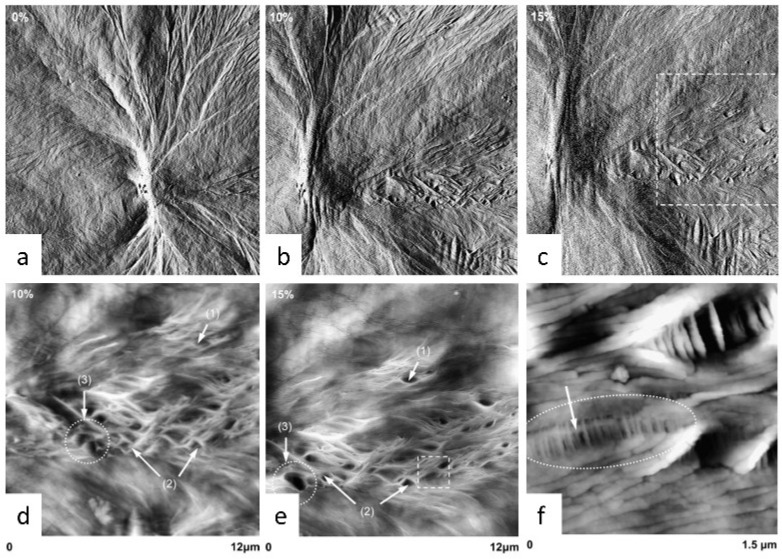
In situ AFM observation of structural changes in i-PB spherulites under tensile stress. (**a**–**c**) AFM amplitude images of spherulites under different strains (0%, 10%, and 15%, respectively). Scan size: 25 × 25 μm^2^. (**d**,**e**) AFM height images (Z-range = 600 nm) from the selected square area of (**c**) for two strain levels, 10 and 15% ((1) the opening, (2) the growth, and (3) the coalescence of cavities are indicated in the images). (**f**) AFM height image (Z-range = 500 nm) from the selected square area indicated in (**e**). Reproduced with permission [90]. Copyright 2007 Elsevier Ltd.

When AFM is integrated with a tensile stage, it is possible to record structural changes in polymer crystals under tensile stress. Thomas et al. used in situ AFM to observe the deformation and crazing of isotactic polybutene (i-PB) spherulites under tensile stress [90]. The tensile testing machine consists of a crosshead that moves via a stepper motor, with the sample clamped at both ends between the moving crosshead and a fixed platform. Before each AFM measurement, the sample needed to be relaxed for 10 min to ensure that it would not move during scanning. Meanwhile, they calibrated the strain of the scanned area structure based on the displacement of the spherulite boundaries. Figure 10a–c show that with stepwise straining from 0% to 15%, the axialite gradually elongated and twisted, accompanied by ridge formation in the equatorial region of the spherulite. The ridged structure was produced by the opening of interlamellar lenticular cavities. Magnifying the observation further reveals that growth and coalescence of holes occurred at the same time, alongside the opening of new holes (Figure 10d,e). Figure 10f provides a clearer view of the internal structure of the cavity. The edges were made up of full lamellae, and numerous microfibrillar structures bridged the two ends inside, which indicates that lamella chain unfolding occurred during stretching, with uniform termination at the interface between crystalline and amorphous on both sides of the craze.

Here, the authors also mentioned that the experimental phenomena they observed were inconsistent with previously reported TEM results [91] and surmised that this discrepancy might be related to the thickness of the i-PB film. Due to TEM testing requirements, the thickness of the i-PB film is restricted to 100~500 nm, resulting in conditions closer to plane stress during stretching. In contrast, the film thickness used for AFM testing was around 100 μm, which more closely approximated triaxial stress stretching conditions. In other words, in situ AFM currently offers unique advantages in monitoring the deformation of bulk samples under triaxial stress.

Subsequently, they investigated i-PB films with smaller spherulites, which had a higher ductility [92]. Unlike large spherulites exhibiting intense cavitation and crazing during tensile, small spherulites fractured more uniformly and showed no tendency for fibrous transformation. This suggests that, while the presence of grain boundaries is expected to lead to stress concentration, stress can effectively be transmitted through these boundaries. Meanwhile, it also indicates that fibrillar transformation is not an inevitable pathway for large strain plastic deformation in semi-crystalline polymers.

Apart from observing the response of crystal structures under external fields, AFM can also directly measure the physical properties of polymer crystals. Currently, piezo force microscope (PFM) testing is widely applied in the field of polymers (especially polyvinylidene fluoride (PVDF), whose β phase exhibits a highly polar arrangement, endowing it with excellent piezoelectric properties [93,94]). The principle involves applying an alternating voltage through conductive probes and utilizing the inverse piezoelectric effect to detect localized deformation in the sample, thereby achieving high-resolution imaging of piezoelectric domain structures. Researchers typically employ PFM to test the piezoelectric properties of modified PVDF materials and establish correlations with their microstructures [95,96,97,98,99]. Additionally, other electrical modes of AFM, such as conductive atomic force microscopy (CAFM) [100], electrostatic force microscopy (EFM) [101], and Kelvin probe force microscopy (KPFM) [102], are also employed to measure the electrical properties of conductive or semiconductive polymers. As AFM technology continues to advance, the range of testing modes is also diversifying. It is important to note that these new methods offer great potential for investigating the structure–property relationships of polymers.

## 6. Application of the Latest AFM Technology

**Figure 11:**
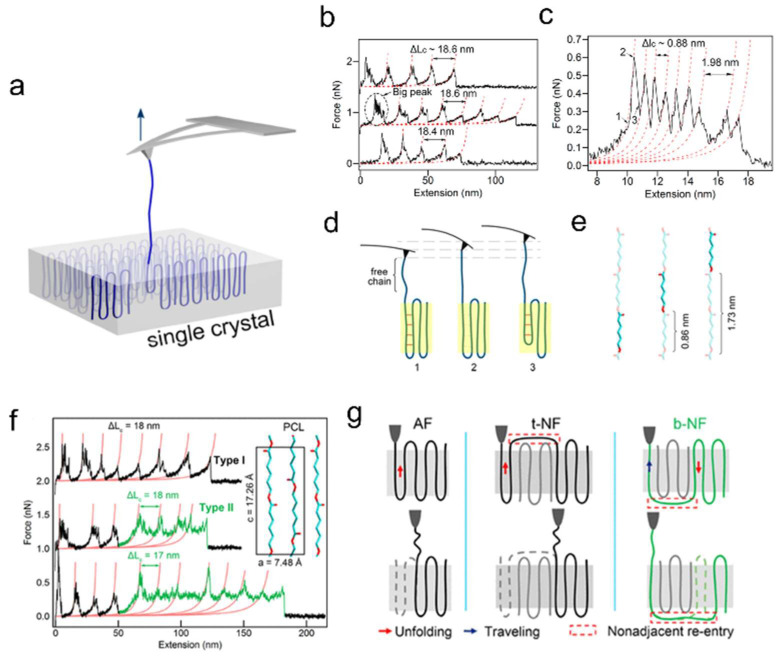
SMFS experiments on a PCL single crystal. (**a**) Schematic of force-induced unfolding of a PCL single chain from a single crystal. (**b**) Force–extension curves and (**c**) an enlarged view of its dashed circle. (**d**) Schematic of the process undergone by the PCL molecular chain during probe pull-off. (**e**) Schematic of PCL repeat unit movement within the crystalline region. Color key: aqua, carbon atoms; red, oxygen atoms. Reproduced with permission [110]. Copyright 2019, American Chemical Society. (**f**) Various types of force–extension curves obtained in PCL single crystals. (**g**) Schematic of the unfolding process of molecular chains with different folding modes. The green color corresponds to the green plateau in part (**f**). Reproduced with permission [108]. Copyright 2019 American Chemical Society.

In addition to serving as an imaging tool, AFM has also been utilized to perform micro-area manipulation of samples. Single-molecule force spectroscopy (SMFS) utilizes probes to manipulate individual molecules and measure forces at the nanoscale, enabling characterization of molecular chain structures (Figure 11a). This technique has found widespread application in both biological and materials science fields [103,104]. Zhang et al. applied SMFS to crystalline polymer systems, utilizing the obtained information to determine molecular chain conformations and folding behaviors within crystals [105,106,107,108,109,110]. They stretched single molecular chains in polycaprolactone (PCL) single crystals, yielding force–extension curves exhibiting two types of sawtooth peaks (large and small ones), with small sawtooth peaks located atop each large peak [110]. The period of the large sawtooth peaks coincided with twice the thickness of the single crystal, corresponding to the unfolding of folds within the single crystal (Figure 11b). For the small sawtooth peaks, their spacing was close to the length of one or two repeat units in a PCL chain (Figure 11c). Therefore, each small peak resulted from the slip of one or two repeat units in a PCL single crystal chain. Figure 11d provides a schematic illustration of the process chains underwent when pulled out of the lattice. First, the free chain segments were straightened, accumulating force within the crystalline region. When the applied force exceeded the dipole interactions between adjacent stems, the molecular chains began to slip within the crystalline region. Molecular chains could opt to slide by the distance of one or two repeat units to form opposite or identical side-chain orientations (Figure 11e).

Furthermore, researchers discovered that PCL single crystals exhibited more than one force–extension curve (Figure 11f) [108]. In view of the strong correlation between these curves and chain folding behavior, they speculated that this was related to irregular chain folding of the single crystal. When a molecular chain underwent nonadjacent folding at the bottom of a single crystal, it had to overcome not only the constraints of the crystal lattice during extraction but also the resistance to chain slip at the crystal bottom (Figure 11g). This resulted in the formation of the green plateau. In other words, using SMFS, they could quantify chain folding in polymer single crystals. Most cases exhibited regular adjacent chain folding, which supports the adjacent re-entry model for solution-grown single crystals. In recent years, SMFS techniques have also been employed to investigate the αc relaxation of stems in PE single crystals [105] and chain tilting at screw dislocations in polyethylene oxide (PEO) crystals [106] and to quantify chain trajectories and long-range correlations within the crystalline–amorphous network of polymer spherulites [111].

In theory, SMFS technology can be applied to any polymer system. However, in practical operation, the coupling between the probe and the molecular chain is critical. If the interaction between the tip and the molecular chain is insufficiently strong, the molecular chain cannot be extracted from the lattice. Liu et al. introduced thiol groups at the terminal ends of PEO molecules [106]. Leveraging Au-S interactions, they used gold-coated tips to randomly adsorb the molecular chains. When the probe was lifted, the corresponding PEO molecular chain was pulled out. For polymers lacking terminal ends, hydroxyl groups can be introduced onto the crystal surface via photooxidation, thereby achieving a similar effect [105].

Moreover, integrating AFM with other testing methods has also been one of the key directions in the development of AFM technology in recent years [16,112]. While AFM can provide information on topography and surface forces, it is incapable of revealing details such as chemical composition and functional group distribution. For this reason, researchers are exploring the integration of AFM with spectroscopic characterization techniques to achieve a one-to-one correspondence between chemical composition and surface morphology.

**Figure 12:**
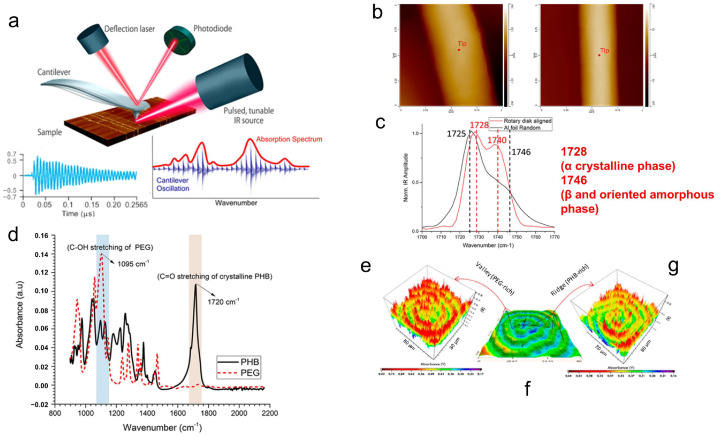
Principles and applications of AFM-IR technology. (**a**) Testing principle of AFM-IR. Reproduced with permission [115]. Copyright 2017, American Chemical Society. (**b**) AFM height images of single electrospun poly(3-hydroxybutyrate-co-3-hydroxyhexanoate fibers collected on aluminum foil (left) and the tapered edge of a rotary disk (right). Scan size: 1 × 1 μm^2^. (**c**) IR spectra of fibers shown in (b). The red dots on the two individual fibers indicate the position of the AFM tip. Reproduced with permission [116]. Copyright 2015, American Chemical Society. (**d**) IR spectra of PHB and PEG. (**e**) AFM-IR mapping of the PHB/PEG (50/50) blend at the 1720 cm^−1^ band. (**f**) AFM height image of the PHB/PEG (50/50) blend. (**g**) AFM-IR mapping of the PHB/PEG (50/50) blend at the 1095 cm^−1^ band. Reproduced with permission [117]. Copyright 2018, American Chemical Society.

Atomic force microscopy–infrared spectroscopy (AFM-IR) is one such example. In traditional infrared spectroscopy instruments, due to the restriction imposed by the diffraction limit, the spatial resolution can only reach the micrometer level at best [113]. But in AFM-IR, its resolution can surpass the diffraction limit, reaching the sub-nanometer level. The principle involves illuminating a sample surface with a tunable, continuous-wave infrared light source. Molecular vibrations, induced by infrared absorption in specific regions, cause transient thermal expansion, which is captured by the probe and converted into cantilever oscillations [114]. The oscillation amplitude of the microcantilever is then used to establish a functional relationship with the infrared wavelength, thereby obtaining the sample’s infrared absorption spectrum [16,115]. After acquiring micro-area infrared spectra, by irradiating the sample with a laser of a specific wavelength, it is possible to generate an infrared absorption distribution map (Figure 12a). Indeed, the spatial resolution tested at this point is no longer determined by the infrared wavelength but by the probe itself—this is why it can overcome the diffraction limit.

Due to its superior resolution, AFM-IR has found extensive application in the analysis of the composition of polymers in micro-areas [118,119,120]. Here, we primarily present two case studies of AFM-IR characterization in polymer crystallization research. The first example aims to highlight that infrared spectroscopy of polymer crystal systems can not only determine chemical composition but also identify crystal structure. Gong et al. characterized electrospun fibers made from poly(3-hydroxybutyrate-co-3-hydroxyhexanoate, PHBHx) with AFM-IR [116]. They used aluminum foil and rotary disks as fiber collectors, respectively, and found that the average diameter of fibers collected by the rotary disks was much smaller than that collected by the aluminum foil (Figure 12b). This indicates that additional stretching likely occurs during formation when using rotary disks for collection. Moreover, they employed AFM-IR to analyze the infrared absorption of single fibers obtained through both collection methods. The results revealed that fibers collected using the rotary disk exhibited stronger infrared absorption at 1740 cm^−1^ (near the characteristic peak of the β and oriented amorphous phases) (Figure 12c), indicating that stretching induced the formation of the PHBHx β phase. This conclusion was also confirmed by wide-angle X-ray diffraction (WAXD) and selected area electron diffraction (SAED) results.

Another example demonstrates the application of AFM-IR in the crystallization of miscible blends. Phuong et al. investigated the three-dimensional sheet assembly and polymer separation mechanisms of equimolar poly(3-hydroxybutyrate) and polyethylene glycol (PHB/PEG (50/50)) at the nanoscale by AFM-IR [117]. Within this system, the infrared characteristic peak spectra of PHB and PEG exhibited distinct differences (Figure 12d), facilitating the analysis of micro-area composition via AFM-IR. Figure 12e shows that in the banded spherulites of the PHB/PEG (50/50) blend, the infrared absorption signal of the C-OH (corresponding to PEG) peak was strong at the valleys but weak at the ridges, while the infrared absorption signal of the C=O (corresponding to PHB) peak exhibited the opposite behavior, being strong at the ridges and weak at the valleys. This might be related to the crystallization sequence of the two components.

However, despite the excellent application potential of AFM-IR in polymer systems, caution must be exercised as the results may lead to the misinterpretation of the information. The interaction between the AFM tip and the sample surface determines the contact resonance frequency of the cantilever. If resonance conditions are not well maintained, for example, at rough surfaces, high noise levels may arise during measurements, or even signal inversion may occur [121]. This means that if handled improperly, AFM-IR results could potentially lead to entirely opposite conclusions. Additionally, when analyzing results obtained from AFM-IR, attention should also be paid to the interference of other factors (such as molecular orientation, crystallinity, thermal expansion coefficient, etc.) with the results. Typically, AFM-IR results require cross-validation with other characterization tests to ensure the reliability of conclusions.

In addition, the coupling of Raman spectroscopy with AFM has also been reported. To overcome the diffraction-limited resolution, Raman near-field scanning optical microscopy (Raman-SNOM) [122] and tip-enhanced Raman spectroscopy (TERS) [123] have been developed. However, their widespread application has not yet been achieved due to the relatively high cost and the requirement for professional handling.

## 7. Summary and Outlook

This paper reviews the application of AFM technology in polymer crystallization research and its unique advantages. In terms of structural characterization, we primarily introduce AFM findings on hierarchical crystalline textures (single crystals, spherulites, shish kebab crystals, etc.), crystal structure, and molecular-scale measurements. Regarding crystallization kinetics, we present in situ AFM measurements of crystallization rates and observations of structural evolution during the crystallization process. As for the structure–property relationships, we focus on characterizing the response of polymer crystals to external fields (heat and force) by AFM and briefly introduce other AFM modes (PFM, CAFM, EFM, etc.) for testing material physical properties. Finally, we also introduce how the latest AFM techniques (single-molecule force spectroscopy and AFM infrared spectroscopy) have aided our understanding of polymer crystalline structures and crystallization processes. In summary, whether from the kinetic or the thermodynamic perspective, AFM holds extensive applications and potential in polymer crystallography.

However, the application of AFM in polymer crystallization research also faces numerous challenges. For instance, the soft surfaces of polymer materials are prone to deformation under probe pressure, yet reducing pressure compromises imaging quality. Furthermore, the crystallization process of polymers is complex and susceptible to external environmental influences. Capturing these dynamic processes accurately and non-invasively via AFM remains a significant challenge.

Nearly half a century has passed since the advent of AFM technology. Today, AFM technology is evolving toward greater precision, efficiency, and versatility. The development of higher-resolution AFM probes and more advanced imaging techniques holds promise for overcoming current limitations. At the same time, AFM is advancing toward integration with other characterization instruments to achieve complementary capabilities. We believe that in the future, AFM will bring greater understanding and discoveries to polymer crystallography.

## Figures and Tables

**Table 1:** Comparison of different characterization methods.

Characterization Methods	Sample Requirements	Crystallographic Information	Advantages	Limitations
X-Ray Diffraction (XRD)	Available in bulk, powder, and film	Crystallinity, crystal form, orientation, etc.	Non-destructive, highly automated, high sensitivity	Low spatial resolution
Differential Scanning Calorimetry (DSC)	Available in bulk, powder, and film	Crystallinity, crystallization (melting), enthalpy, etc.	Highly automated, capable of quantitative analysis	Low spatial resolution, destructive
Optical Microscopy (OM)		Morphology	Simple operation, wide range of applications, non-destructive	Resolution limited to the micrometer level
Scanning Electron Microscopy (SEM)	Must be conductive (non-conductive samples require gold or carbon spraying to enhance conductivity)	Morphology	High spatial resolution (around 1 nm), large depth of field, minimal electron damage	Vacuum test environment, low resolution in the vertical direction
Transmission Electron Microscopy (TEM)	Small (less than 2 mm in width) and thin (less than 500 nm in thickness), generally requires special processing (deposited on copper mesh, ultrathin sectioning, or surface replica)	Morphology, crystal structure	Extremely high spatial resolution (better than 0.2 nm)	Vacuum test environment, high electron damage, difficulties in sample preparation
Atomic Force Microscopy (AFM)	Surface roughness less than 1 μm	Morphology, physical properties (modulus, electrical conductivity, piezoelectricity, etc.)	Almost non-destructive, high spatial resolution (vertical: better than 0.1 nm; horizontal: depends on the probe diameter, typically around 1 nm), can be tested in gas, liquid, and vacuum environments	Slow scanning speed, size limitation, operational experience required

## Data Availability

No new data were created or analyzed in this study.
